# First Report of CRISPR/Cas9 Mediated DNA-Free Editing of *4CL* and *RVE7* Genes in Chickpea Protoplasts

**DOI:** 10.3390/ijms22010396

**Published:** 2021-01-01

**Authors:** Sapna Badhan, Andrew S. Ball, Nitin Mantri

**Affiliations:** The Pangenomics Lab, School of Science, RMIT University, Melbourne 3000, Australia; s3418286@student.rmit.edu.au (S.B.); andy.ball@rmit.edu.au (A.S.B.)

**Keywords:** CRISPR/Cas9, chickpea, DNA free gene editing, protoplast, *4-coumarate ligase* (*4CL*), *Reveille 7* (*RVE7*), drought stress

## Abstract

The current genome editing system Clustered Regularly Interspaced Short Palindromic Repeats Cas9 (CRISPR/Cas9) has already confirmed its proficiency, adaptability, and simplicity in several plant-based applications. Together with the availability of a vast amount of genome data and transcriptome data, CRISPR/Cas9 presents a massive opportunity for plant breeders and researchers. The successful delivery of ribonucleoproteins (RNPs), which are composed of Cas9 enzyme and a synthetically designed single guide RNA (sgRNA) and are used in combination with various transformation methods or lately available novel nanoparticle-based delivery approaches, allows targeted mutagenesis in plants species. Even though this editing technique is limitless, it has still not been employed in many plant species to date. Chickpea is the second most crucial winter grain crop cultivated worldwide; there are currently no reports on CRISPR/Cas9 gene editing in chickpea. Here, we selected the *4-coumarate ligase* (*4CL*) and *Reveille 7* (*RVE7*) genes, both associated with drought tolerance for CRISPR/Cas9 editing in chickpea protoplast. The *4CL* represents a key enzyme involved in phenylpropanoid metabolism in the lignin biosynthesis pathway. It regulates the accumulation of lignin under stress conditions in several plants. The *RVE7* is a MYB transcription factor which is part of regulating circadian rhythm in plants. The knockout of these selected genes in the chickpea protoplast using DNA-free CRISPR/Cas9 editing represents a novel approach for achieving targeted mutagenesis in chickpea. Results showed high-efficiency editing was achieved for *RVE7* gene in vivo compared to the *4CL* gene. This study will help unravel the role of these genes under drought stress and understand the complex drought stress mechanism pathways. This is the first study in chickpea protoplast utilizing CRISPR/Cas9 DNA free gene editing of drought tolerance associated genes.

## 1. Introduction

CRISPR/Cas9 based gene editing has revolutionized targeted gene editing in plants in recent years [1,2,3,4,5,6,7,8,9,10]. The CRISPR/Cas9 method of gene editing is based on a natural immune system used by bacteria to prevent infection by viruses [11]. However, recently this mechanism was used for genetically editing DNA of interest. CRISPR/Cas9 genome editing has broad application in crop improvement and can be used to develop designer genetically edited non-GM crops. A key focus of agricultural scientists is the implementation of this approach to plant breeding for the development of new varieties of crops with higher tolerance to environmental constraints [11,12]. The most recent approach to genetic engineering is genome editing by programmable endonucleases. In CRISPR/Cas9 gene editing, Cas9 endonuclease enzyme is explicitly used for inducing double strand breaks in target genes of interest. However, to restore the damage by the double-strand break, the cellular DNA repair pathway then acts through non-homologous end joining (NHEJ) or homology-directed repair (HDR) systems. Insertions, deletions, base substitutions, and DNA recombination can occur in the repair mechanism process, leading to a frameshift of the sequence [13,14].

CRISPR/Cas9 gene-editing tools have been utilized for gene activation, repression, knockout, knockdown, repression, and for altering epigenetic modifications in several plants crops such as Arabidopsis [15], apple [8], citrus, carrot [16], grape [17], tomato [18], rice [19], sorghum [20], maize and soybean [21], and wheat [22]. However, gene free editing has effectively been achieved in only a few crop varieties such as Arabidopsis [23], bread wheat [4], grapevine, and apple [6]. For example, CRISPR/Cas9 genome editing was used to identify abiotic stress response in Arabidopsis plants; the results suggested that OST2 (proton pump), a mutant allele obtained from editing, altered stomatal closing under environmental stress [8]. Another recent study in maize used the CRISPR/Cas9 system to produce novel allelic variations which could be used for breeding drought-tolerant crops. ARGOS8, whose overexpression can lead to reduced ethylene sensitivity, was genetically modified by this system and field studies revealed that ARGOS8 variants had increased grain yield under drought stress; further, no loss in yield was recorded under well-watered conditions [24].

Global climate change and population increase have put pressure on the agriculture industry to improve productivity, leading to the development of new, improved technologies associated with enhancing the ability of crops to continue to be productive under sub-optimal conditions such as elevated temperature and reduced moisture availability. There remains an urgent need for DNA-free gene editing because there are always chances of foreign DNA integration using plasmid-mediated approaches to gene editing [25]. Current genetically modified organisms (GMO) regulations are stringent, making it challenging to produce commercial genetically modified food crops [10].

Due to its high nutritional value chickpea is the second largest internationally produced cool-season food legume. To date, a low degree of intraspecific genetic diversity has limited success in increasing the yield and quality of cultivated chickpeas. Croser et al. proposed that increased genetic diversity can be achieved from within the forty-three species of the *Cicer* genus by hybridizing the cultivated species with unimproved ‘wild’ relatives to integrate beneficial traits from the eight species that share an annual growth habit and chromosome number with cultivated chickpea. Furthermore, the morphological characteristics and tolerance to a variety of abiotic and biotic stresses of potential advantage to chickpea improvement programs have been identified in the screening process [26]. Consequently, for the development of transgenic chickpea plantlets derived from embryonic axis co-cultivation, Agrobacterium-mediated transformation and other standard protocols have been developed [27,28]. However, only a few reports on the use of genetic transformation/transgene(s) for the development of abiotic stress tolerance transgenic chickpea plants have been published [29,30,31]. Transgenic chickpeas have been developed based on the insertion of the abiotic stress-tolerant bacterial codA gene. Anwar et al. concluded that the application of genomic techniques for the analysis of the chickpea genome and improvement of chickpea would be significantly facilitated by the development of a robust and reproducible genetic transformation method [27]. Chickpea CarNac3 transgene showed increased drought tolerance in poplar plants [32]. Hajyzadeh et al. identified that the overexpression of miR408 leads to drought tolerance in chickpea [33]. One of the significant constraints in terms of chickpea production is drought stress. Many previous studies have focused on this issue using genetic engineering of chickpea cultivars [30,31,32,33,34,35]. However, recent innovations in in vitro culture and gene technology give unique opportunities to the full potential of the cultivation of chickpeas using these new technologies. Regrettably, no transgenic chickpea variety in the world has been approved for cultivation [36].

In this study, two potential drought tolerance genes were selected for transformation in a commercial Kabuli chickpea genotype. The *4-coumatrate: CoA ligase* gene codes for coumarate ligase enzyme which is well known for its role in the biosynthesis of secondary plant metabolites during phenylpropanoid metabolism and in major branch pathways [37,38,39]. This phenylpropanoid enzyme is essential for the activation of the hydroxycinnamic acids during lignin biosynthesis. A study in Arabidopsis showed that a decrease in *4CL* activity correlated with a reduction in thioglycolic acid extractable lignin [38]. Further, the increased expression of the lignin biosynthesis gene was observed in watermelon during water stress [40].

*RVE7* is a gene that encodes the transcription factor involved in circadian rhythm and the opening of cotyledon mediated by phytochrome A. The regulation of the *RVE7* gene is controlled by *LHY* and *CCA1* central oscillator mediation. It is known to regulate its expression and belongs to part of the circadian feedback [41]. *RVE7* is active in controlling the circadian clock’s downstream processes such as hypocotyl growth and flowering [42]. Transcription factors such as *REVEILLE 2* (*RVE2*), *RVE7*, *RVE8*, and *MYB HYPOCOTYL ELONGATION-RELATED* (*MYBH*) act in ways like *CCA1* and *LHY1*.1,3,4,6,9–14 on the circadian clock. *RVE7/EARLY-PHYTOCHROME-RESPONSIVE1* (*EPR1*) was found to be phytochrome A and phytochrome B regulated and to work as a slave oscillator part [42,43]. Studies of other clock related MYB proteins demonstrated that these variables have a less prime role in regulating *TOC1* expression. *RVE7/EPR1* does not control the expression of *CCA1*, *LHY*, or *TOC1*, although these oscillator components are involved in the circadian regulation of *RVE7/EPR1* [44].

**Figure 1:**
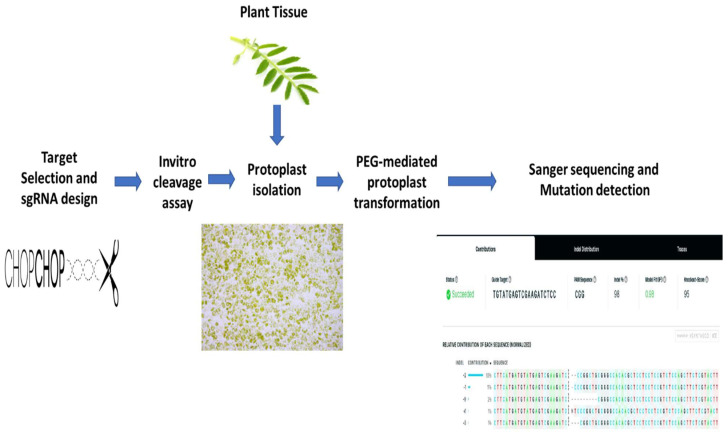
Graphical abstract: An overview of the CRISPR/Cas9 DNA free gene editing in the chickpea protoplast. The protoplasts of young leaves were isolated and transformed with CRISPR RNP complex under optimum conditions. The DNA was extracted from the protoplast control and test samples and sent for Sanger sequencing to detect the mutations. The transformation efficiency and knockout score were identified using ICE bioinformatics tool (Synthego).

Here, a pilot study was conducted to knockout the *4CL* and *RVE7* genes in the chickpea protoplast to help understand the drought response mechanism in chickpea. The main aim of this study was to conduct CRISPR/Cas9 for the polygenic adaptive trait (i.e., drought tolerance) and propose the method for the use of DNA free gene editing in recalcitrant species. Figure 1 provides an overview of CRISPR/Cas9 gene-editing process and how it was employed in this study. 

## 2. Results

### 2.1. sgRNA Selection and Design

**Figure 2:**
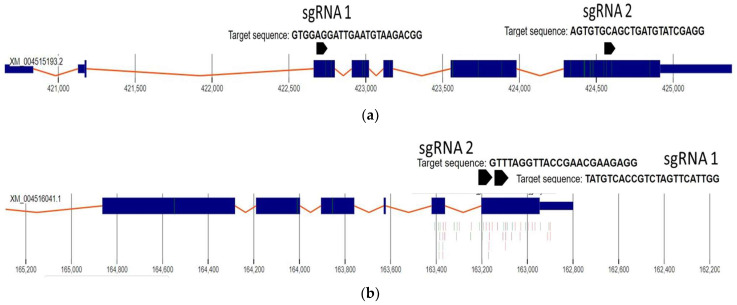
Systematic design to show the location of sgRNA target sites in *RVE7* and *4CL* nucleotide sequences. (**a**) Systematic illustration of the nucleotide sequence of *RVE7* gene locus. (**b**) Systematic diagram of the nucleotide sequence of *4CL* gene locus. The blue boxes represent exons and orange connecting lines represent introns.

Two candidate genes (*4CL* and *RVE7*) which are potentially associated with drought tolerance in chickpea were selected for the knockout in chickpea protoplasts. The sgRNA for the target location were designed using CHOPCHOP and verified by other tools such as CCTop using the genome sequence for Kabuli chickpea. The preferred sgRNA based on the target selection algorithm were selected for further studies. The illustration of the location of sgRNA target sites in *RVE7* and *4CL* nucleotide sequences is shown in Figure 2.

### 2.2. sgRNA Selection and Design In Vitro Digestion Assay

**Figure 3:**
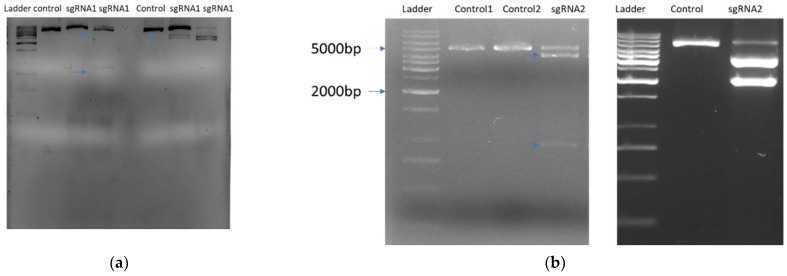
Results of in vitro digestion cleavage assay of both sgRNA sets for *4CL* and *RVE7* gene. (**a**) sgRNA 1 for *4CL* and *RVE7*. The DNA of 5 kb PCR-amplified fragments for gene *4CL* and *RVE7* were treated with preassembled RNPs (sgRNAs (crRNA/tracrRNA) + Cas9) and in vitro cleavage assay was performed. For in vitro cleavage assay non-treated samples were used as negative controls in gel electrophoresis. (**b**) sgRNA 2 for *4CL* and *RVE7*. The digested samples for 4CL left side and *RVE7* at right side. Above lane 2,3,4 correspond to *4Cl* and lane 6,7,8 for *RVE7* samples. The expected band size for sgRNA 1 *RVE7* were approximately 2298 and 3543; sgRNA 2 4469 and 1363. For *4CL* the expected band sizes after digestion for sgRNA1 were 4931 and 1170; sgRNA 2 approximately 4477 and 1428.

The primary step for the CRISPR/Cas9 gene editing is the selection of sgRNA sequences for target knockout. Two sets of sgRNA were designed using CHOPCHOP and were tested in vitro for their efficacy. In order to determine the specificity of the target sgRNA sequences, a 5 kb region around the target regions were amplified. Primers were designed to amplify the target region for both target genes and in vitro cleavage assay was conducted using the respective purified PCR products and preassembled RNPs. The in vitro digestion shows that individual band sizes were achieved with both sgRNA sets in *RVE7* and *4CL* amplified fragments (Figure 3). The sequences used for the amplification of DNA and position of the sgRNA sequences are provided in Supplementary Material 1 and 2.

### 2.3. Protoplast Isolation and Transformation

**Figure 4:**
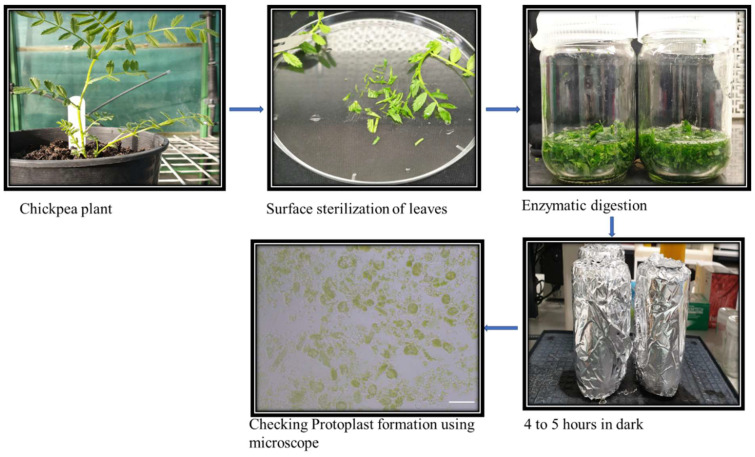
Flow chart of the steps performed during the protoplast isolation from leaf tissue of chickpea plants. The leaves from the 4 to 5 weeks old plants were collected and surface sterilized. The leaves were cut into small pieces. The enzyme solution was prepared and cut leaf sections were kept in the dark for 4 to 5 h digestion resulting in protoplast formation. After 2 h, samples for protoplast formation were collected to check the protoplast formation stage, and once the protoplast were isolated, they were used for the PEG mediated transfections.

Protoplasts were isolated using the BioWORLD kit with minor modifications. The protoplast was isolated from young chickpea leaves from 4 to 5-week-old plants (Figure 4). The viability and protoplast number were lower initially but optimized by changing the leaf maturity, amount of leaves used, centrifugation speed, and time for the enzymatic digestion.

### 2.4. Mutation Detection

**Figure 5:**
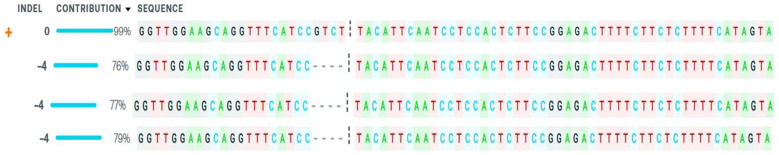
Illustration of ICE Analysis of *RVE7* sgRNA1 (GTGGAGGATTGAATGTAAGACGG) RNP complex edited protoplast cells in chickpea. The respective protoplast samples were edited and sequenced with Sanger sequencing (Macrogen, Seoul, Korea) and further analyzed with the ICE (Synthego, Redwood City, CA, USA) online tool to deconvolute the pooled protoplast DNA. The contributions show the sequences inferred in the edited protoplast population and their relative proportions. The “+” sign is for the wild type sample. The target cut sites are denoted by black vertical dotted lines. The trace plots for the edited samples below show the cut site and protospacer adjacent motif (PAM) sequence labelled in the edited sample and control sample. The sgRNA 2 sequencing samples had low quality before the cut site and could not be analyzed using the ICE tool.

**Figure 6:**
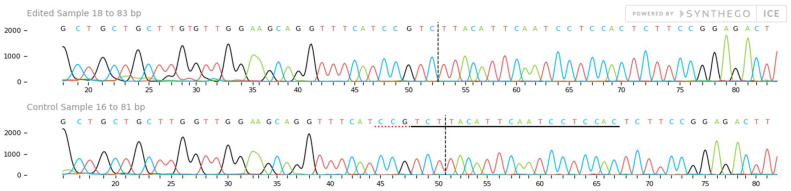
Traces of the edited and control population of the protoplast as shown by the Sanger Scheme 1. files control and the experimental study, which contain mixed base call. The region underlined in horizontal black represents the guide sequence. The PAM site is represented as the horizontal red focus. The vertical dotted black line represents the site of the actual break. Cutting and fixing which is sensitive to errors usually results in mixed sequencing bases after cutting.

Sanger sequencing was performed to detect mutations in the transfected protoplasts. The DNA was isolated from the transformed protoplast, and target regions were amplified using primer sets to conduct sequencing. The PCR program for sequencing samples is also provided in Table S2 and the PCR reaction to amplify the sequencing fragment is provided in Table S4. The results obtained from Sanger sequencing are classically given as nucleotide occurrence chromatograms for respective samples, represented as distinct colored peaks. Usually, the biggest drawback in protoplast mutation identification is that mutated protoplast DNA chromatograms do not provide any apparent indication of the mutation due to the difference in editing forms in each protoplast cells and the presence of unmutated protoplast cell DNA in each sample. To overcome these challenges, Inference of CRISPR Edits (ICE), a bioinformatics tool by Synthego, was used for mutation detection [45] The *RVE7* sgRNA 1 results showed 76%, 77%, and 79% indel percentage for the treated protoplast samples compared to the 99% for control sample, with average of 77.3% and standard deviation of 0.81 for all treated samples (Figure 5 and Figure 6). Indel percentage is the editing efficiency (percentage of the pool with non-wild type sequence) as determined by comparing the edited trace to the control trace. The knockout score represents the proportion of cells that have either a frameshift or 21+ bp indel. The knockout score is used in an understanding of the number of the contributing indels expected to result in a functional knockout of the target gene.

**Figure 7:**
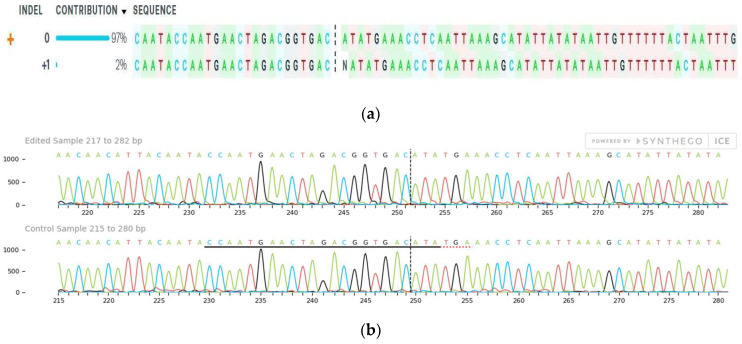
ICE results for *4CL*. (**a**) The sequence plot represents the ICE Analysis of *4CL* sgRNA1 Target 1 (sgRNA:CCAATGAACTAGACGGTGACATA) RNP edited protoplast cells in the chickpea. The contributions show the sequences inferred in the edited protoplast population and their relative proportions. The cut sites are represented by black vertical dotted lines and “+” far left side denotes the control sample. (**b**) The trace plot of the *4CL* sample 4 edited and control population of the protoplast showing the PAM sequence and cut site.

The sequencing samples for *4CL* sgRNA 1 were also analyzed using the ICE tool. However, in *4CL* samples only one sample showed +1 indel with knockout score of 2%, shown in sequence and trace plot (Figure 7). Nevertheless, all other *4CL* test samples showed no indel percentage. The sgRNA 2 sequencing results were low quality and did not fit the threshold values for ICE software.

## 3. Discussion

The simplicity of the CRISPR/Cas9 system offers agricultural scientists an opportunity to edit a gene of interest at almost any laboratory-based facility effectively and economically. Successful DNA-free editing with the CRISPR/Cas9 system is important for crops such as chickpea, due to the crop’s high demand and export value but low productivity because of challenging climatic conditions. The main drawback of plasmid-mediated delivery in genome editing is the possibility of random integration of plasmid sequence in the host genome. In addition, the current GMO regulations and the complication of commercialization of genetically edited varieties makes DNA-free gene editing more suitable compared to traditional gene editing techniques [6]. It will help to grow crops that are resistant to different biotic and abiotic stresses if the CRISPR/Cas9 technique can gain social acceptance and still comply with the strict regulations set by global and local authorities. Moreover, other techniques used for gene editing, due to the random nature of the gene integration such as random mutagenesis, are not able to control every gene in the process. With the aid of precise genes used by the CRISPR/Cas9 method, genes can be altered in crops without the introduction of a transgene [11].

However, while the CRISPR/Cas9 system appears to be extremely promising in many crop improvement areas and might even be omitted from the current GMO legislation, there are some factors that need to be noted. To acquire social acceptance of DNA-free genome editing, social impact understanding is very critical because society needs to understand how the method functions. It is a necessity to educate society that the final products from this technique are not considered GMOs [11,45]. An obstacle to this is the confusion about possible side effects on modified crops. Moreover, it is clear that the potential this system has for the world’s food supply scarcity will largely depend on the society’s perception of the crops that have been modified with CRISPR/Cas9 [46].

Due to limited CRIPSR editing studies conducted in legumes, although it is difficult to achieve high-quality results, it is a necessary journey of discovery. Due to technical complications in developing stable transgenic chickpeas and the absence of a steady transient system of expression for rapid analysis of gene expression and function, there is a requirement to conduct further studies in this area. To our knowledge, this is the first study to achieve the knockout of two drought tolerance associated genes in chickpea protoplast.

The viability of the isolated protoplast was lower when older leaves were used for protoplast isolation compared to young leaves. It is important to check the viability of the protoplast during the transfection experiment. Cheng and Nakata in a recent study suggested that viability of the isolated chickpea protoplasts should be monitored at different steps in the transfection process [47]. Here, in this study, the Sanger sequencing results showed higher editing efficiency for the *RVE7* gene compared to the *4CL* gene. It was observed that even if the sgRNA selected for the knockout works effectively during the in vitro digestion assay, it does not necessarily translate to reliable in vivo editing of the gene. Out of the two sgRNAs selected for the knockout, only one showed in vivo effectiveness. Recently, a study in *N. benthamiana* to evaluate sgRNA efficiency using different online tools found no significant correlation between the predicted rankings and the editing frequencies in vivo in the transformation of *N. benthamiana* compared to in vitro CRISPR digestion [48]. Further, a study in wheat showed that the PinB-D gene had 0% editing efficiency in wheat protoplasts; however, the sgRNA showed optimum results during in vitro digestion [49]. Here, the *4CL* protoplast sample only showed a 2% knockout score which may be due to sequencing artefacts or the presence of a target site in the conserved domain of the gene which made access to the RNP complex difficult. Therefore, the most likely reason for unedited protoplast cells could be the inaccessibility of the target site by the RNP complex in the in vivo environment. Amongst the various limitations of the CRISPR/Cas9 gene editing in plants, the most significant is a recalcitrant sgRNA/target site due to chromatin conformation [48,50]. A study in cucumber reported the occurrence of chromatin structural rearrangements during the de-differentiation of protoplasts with no obvious change in the expression profile [51]. Studies have confirmed that certain sgRNAs may show low efficiencies or may even fail to work in many setups, which may occur due to the chromatin states of the target loci, unwanted hairpin structures of selected sgRNA, or additional unidentified factors [52]. In this study, the main objectives were to set up a CRISPR/Cas9 system for chickpea to knockout two potential drought tolerance candidates using protoplast cells in vivo. In mammalian cells, many studies have shown that conformation of the chromatin surrounding a sgRNA/Cas9 target site may significantly affect the site’s accessibility to the RNP complex [50,53].

Interestingly, in plants, the tools used to design the sgRNA does not take the chromatin conformation into account when predicting sgRNAs because of the lack of knowledge of the epigenetic profiling in many plant genomes [48]. In addition, the secondary structures of sgRNAs are proposed to have a critical consequence for Cas9/sgRNA effectiveness in many studies [54,55,56]. A study in rice reported relatively higher editing efficiencies in target sequences with greater GC content. It is therefore desirable to pick targets with a GC content between 50 and 70%, and those with less or no base pairing with the sgRNA sequence. The use of higher GC content target sequences may theoretically contribute to a greater risk of off targeting [57]. A study in rice reported reduced editing efficiency of the *Os07g0261200* gene due to the low GC content (35%) of the target sequence [58].

Further, the inaccessibility of the RNP complex in the *4CL* target site may also be due to the biological function of this gene. Generally, the *4CLs* are known to play a vital role in generating Coenzyme A (CoA) esters within the phenylpropanoid pathway. This channels carbon flow into various secondary phenolic metabolism branching pathways, producing different groups of secondary natural phenolic products, including flavonoids, lignin, suberins, and coumarins, which play an essential role in the growth of plants and environmental interactions [59,60]. Hu et al. proposed that the lignin content in maize leaf can play an important role in drought tolerance and needs to be further explored as a molecular marker for drought tolerance traits [61]. A recent study in cotton by Sun and others suggested that *Gh4CL7*-silencing amplified sensitivity to drought stress. However, the overexpression enhanced drought tolerance, which indicates the function of *4CL* genes and their potential role in drought tolerance [62]. Another recent study in chickpea identified metabolic pathways that are differentially regulated in different genotypes; for example, phenylpropanoid metabolism, and biological processes such as stomatal development with potential consequences for drought tolerance [63]. *RVE7* and other family members are well known for their role in the circadian clock, which coordinates biological processes related to daily and seasonal environmental changes [42,64]. The information available so far suggests the importance of exploring the roles of these candidate genes to establish the basis of drought tolerance in chickpeas. The drought tolerance pathways play a significant role in the activation of signaling mechanisms and differentially expressed genes. The manipulation of the novel candidate genes identified may help to achieve the drought resilient chickpea varieties. Climate change has already affected many crops worldwide, but it is expected to go beyond the drought tolerance and will impact mineral nutrient availability and accumulation to the plants and soil. It is predicted that the microorganisms will be affected by global climate which will further affect the plant-soil interactions [65]. The protoplast transformation method presented here in chickpea can be utilized as a screening protocol for in vivo efficiency of the selected sgRNAs for a target site. This will save not only time but also resources, avoiding the selection of sgRNAs which may not be accessible for in vivo editing. Confirmation of the target editing in the protoplast can be set closer to the actual editing in plants. The RNP complex for *RVE7* designed in this study can be used in future for producing CRISPR-edited chickpea plants and validating the physiological role of this gene in chickpea.

## 4. Conclusions

This study is the first proof of concept study which shows that CRISPR/Cas9 gene editing can be employed to achieve targeted genome editing in chickpea protoplasts. The results obtained from this study could help in developing new traits and understanding the drought mechanism in chickpea plants by knocking out the desired gene, followed by protoplast regeneration or using the plant tissue for transformation. In several plant species the transient expression systems have been demonstrated to be helpful in rapid high-throughput screening and systematic characterization of interesting genes and proteins. Such transient expression systems are particularly important in plants where stable transformation protocols are time-consuming and inefficient. The transient chickpea expression system using protoplasts presented in this study can be used as an effective method to help harness the cumulative amounts of existing genomic data resources and can also be used to speed up the discovery of genes that survive drought and improve crop characteristics in this essential legume crop. The functional validation of the genes studied in this study should be performed to understand their individual role in the drought tolerant genotypes. The particular effect of the successful knockout under drought stress imposition needs to be further studied. As fewer studies have been conducted on the role of these genes in the drought mechanism, establishing more stringent experiments using NGS and qPCR for expression profiles and microscopy to examine the structural changes will be of great importance for the development of new chickpea varieties.

## 5. Materials and Methods

### 5.1. Chickpea Plant Material and Cas9 Protein

Commercial Kabuli chickpea plants were grown in a glasshouse facility at RMIT Bundoora campus. The 1.5 g leaf tissue from young chickpea plants was collected for the protoplast isolation. The recombinant *S. pyogenes* Cas9 nuclease (Integrated DNA Technologies, IDT, Coralville, IA, USA), purified from an *E. coli* strain expressing the nuclease was used in this study. It consisted of a nuclear localization sequence (NLS) and C-terminal 6-His tag, which is provided in solution at 10 µg/µL. The components of the crRNA:tracrRNA duplex were Alt-R^®^ CRISPR-Cas9; crRNA contained the target-specific sequence for guiding Cas9 protein to the genomic location of the selected target sequences, and Alt-R^®^ CRISPR-Cas9 had tracrRNA which hybridizes to crRNA to activate the Cas9 enzyme. The required dilution was performed according to the manufacturer’s protocol under controlled conditions.

### 5.2. Target Site Selection and sgRNA Design

Two potential drought tolerance associated genes (*4CL* and *RVE7*) were selected for knockout based on their expression levels in ICC283 (drought sensitive genotype) and ICC8261 (drought tolerant genotype) under drought stress. The expression levels for the selected genes are shown in Table 1.

The sgRNA targets were designed using CHOPCHOP tool [66]. CHOPCHOP can predict the frameshift rate of each gRNA which are target sites for high-efficiency editing. All the recommended parameters were selected for the range of sgRNA sequences; for traditional Cas9 (20bp-NGG), preferred features for effective sgRNA include: gRNA has no off-targets; gRNA is expected to target all isoforms of the target gene and gRNA downstream of any in-frame ATG to avoid the expression of truncated proteins. Based on these selection criteria, the top sgRNA were selected for the knockout experiments. The sgRNA used in this study are shown in Table 2 below.

### 5.3. In Vitro Cleavage Assay

The 5 Kb DNA fragments for selected target genes containing the target site were amplified using PCR, purified, and eluted with RNase-free water. The RNP complex was prepared according to manufacturer’s instructions and 1 μM RNP was mixed with the purified target DNA (100–150  ng) in 10 X Cas9 reaction buffer (20 mM HEPES, pH 7.5, 150  mM KCl, 10  mM MgCl_2_, 0.5  mM DTT) in a total volume of 10 μL, followed by digestion at 37  °C for 60 min. Further, to release the DNA substrate from the complex, 1 μL proteinase K was added to reaction and incubated for 10 min at 56 °C (https://sg.idtdna.com/pages/support/guides-and-protocols). The digested DNA products for respective samples were separated on a 2% agarose gel immediately after the digestion process, and cleavage activity was measured by the number of digested products over the total amount of input target DNA. The obtained DNA bands were quantified using Gel quantification software (ChemiDoc imaging system, BIO-RAD, Hercules, CA, USA). The in vitro digestion assay was performed before every transformation to confirm that the RNP complex was functional and capable of editing the respective genes.

### 5.4. Protoplast Isolation

Leaf tissue from Kabuli chickpea was collected to produce protoplasts using a Plant Media Protoplast Isolation kit (PlantMedia™, a division of bioWORLD, Visalia, CA, USA, Catalog no. SKU# 30210002-1) using a slightly modified protocol. Briefly, 1.5 g fresh plant leaf tissue was surface sterilized by 2% bleach followed by three distilled water washes for 5 min each. The leaf tissue was sliced thinly and resuspended in distilled water in 50 mL tubes and centrifuged at 300× *g* in a swinging bucket rotor for 10 min at room temperature. Suspended cells were washed with 5 mL cell wall wash buffer. Pellets were resuspended in 5 mL of ice-cold protoplast enzyme solution and transferred to a 25 mL flask. The flasks were kept at room temperature for 5 h in the dark with gentle agitation at 100–150 rpm. The protoplast formation was checked in using 10 μL of suspension on a cell counter and microscope. The protoplast suspension was centrifuged in 50 mL tubes at 300× *g* for 5 min at 4 °C. The protoplasts were washed twice by resuspending the pellet in 5 mL of ice-cold protoplast wash solution and centrifuged at 300× *g* for 10 min at 4 °C. Finally, BSA (0.5 mg/mL) was added to the final protoplast preparation for transformation. The viability of the protoplast was calculated via a cell counter (Invitrogen™ Countess™ Automated Cell Counter) and 2 × 10^5^ cells used for transformation.

### 5.5. Protoplast Transformation with RNP Complex

A polyethylene glycol (PEG4000)-mediated transformation was used to transform respective RNP complexes into chickpea protoplast cells. The PEG transformation protocol for the efficient targeted knockout in both genes was adapted from previous studies [6,10,49,67]. The 2 × 10^5^ resuspended protoplasts were transformed with Cas9 protein and sgRNA in a ratio of 1:1. Protoplasts (200 μL, 2 × 10 cells) and RNPs for example, 1:1 is Cas9 30 μg (stock 10 μg/μL) and sgRNA 30 μg (stock 10 μg/μL) used for transformation. Before the transformation experiment, Cas9 and sgRNA were pre-mixed to form the RNP complex and incubated at room temperature for 10 min. Further, all the components, protoplast, Cas9, and sgRNA mix were combined, and an equal volume of freshly prepared PEG 4000 added (40% PEG [*w*/*v*] PEG 4000, 0.2 M mannitol, 0.1 M CaCl_2_), with immediate mixing to prevent aggregation. This mixture was incubated at room temperature for 20 min. An aliquot (400 µL) of W5 solution (2 mM MES pH 5.7, 154 mM NaCl, 125 mM CaCl_2_ and 5 mM KCl) was added, mixed, and incubated at room temperature for 10 min. An additional 800 μL of filter sterilized W5 solution was added to the tubes, mixed gradually, and incubated at room temperature for 10 min. The tubes were centrifuged at 50× *g* for 5 min; the supernatant was discarded, and another 1 mL of W5 solution was added to the tubes. Subsequently, the tubes were incubated overnight at room temperature in the dark. The lower sediments were collected for genomic DNA isolation from the protoplast cells. Three biological replicates of the protoplast transformation were performed for each set of genes. Genomic DNA was isolated from protoplast cells for Sanger sequencing. The sample treated only with the Cas9 enzyme was used as a negative control. The transformation experiments were repeated twice, and similar results were obtained both times.

### 5.6. DNA Extraction and PCR Amplification of Target Regions

Genomic and protoplast DNA were extracted using Isolate II Genomic DNA kits (Catalog number: BIO-52069, Bioline (Aust) Pty Ltd., Eveleigh, Australia). The DNA concentration was determined with a Nanodrop spectrophotometer (Thermo Scientific, Waltham, MA, USA); the concentration of the samples was around 25–30 ng/μL. The PCR reaction to amplify the 5 kb genomic region and program used is provided in Table S1 and the PCR reaction for sequencing samples and program are also provided in Table S3. The 5 kb fragment was amplified using GoTaq^®^ Long PCR Master Mix (Catalog number: M4021, Promega, Alexandria, NSW, Australia). The samples for sequencing the target regions were amplified using MyTaq™ Red Mix, 2× (Catalog number: BIO-25043, Bioline (Aust) Pty Ltd.). The primer used for amplification of CRISPR target loci of *4CL* and *RVE7* are presented in Table 3 and the PCR reaction to amplify the sequencing fragment is provided in Table S4.

## Figures and Tables

**Table 1:** The gene expression values of the *RVE7* and *4CL* in both genotypes. Shoot apical meristem (SAM), flowering bud (FB), fully opened flower (FOF), young pod (YP), partially open flower (POF).

Gene Name	ICC8261 (Drought Tolerant Genotype) Expression Levels	ICC283 (Sensitive Tolerant Genotype) Expression Levels e 3
*RVE7*	SAM 2.69, FB-3.4, POF0, FOF-1.29, YP1.9	SAM 3.4, FB-3.02, POF0, FOF-5.9, YP 0
*4CL*	FB 10.39	Not Differentially expressed data

**Table 2:** List of sgRNAs designed to target *4CL* and *RVE7* genes in chickpea. A 20 bp sequence of crRNA/tracrRNA with PAM in red.

Gene Name	sgRNA Name	sgRNA Sequence
*4-coumarate-CoA ligase-like 1* NW_004516753.1_162798-165892 Length 3094	*4CL*sgRNA1 *4CL*sgRNA2	TATGTCACCGTCTAGTTCATTGG GTTTAGGTTACCGAACGAAGAGG
*REVEILLE 7-like* NW_004516329.1_420654-4253847	*RVE7*sgRNA1	GTGGAGGATTGAATGTAAGACGG
	*RVE7*sgRNA2	AGTGTGCAGCTGATGTATCGAGG

**Table 3:** The sequences of primer used for amplification of CRISPR target loci of *4CL* and *RVE7*.

Primer Sets	Sequence	Expected Product Size
*4CL* Primer Set1	Forward: ACAATACCAATGAACTAGACGGTGA Reverse: TCCCTAACAAAATCCAACACATCT	592
*4CL* Primer Set2	Forward: ACAATACCAATGAACTAGACGGTG Reverse: TCCCTAACAAAATCCAACACATC	590
*RVE7* Primer Set1	Forward: AACATGCTGCTGCTTGGTTG Reverse: GACGAAGAGAGGGACTAATTTCA	398
*RVE7* Primer Set2	Forward: GGAAGCAGGTTTCATCCGTC Reverse: TGATGAAAGAAATTGATGCTCACTA	420

## Data Availability

The data that support the findings of this study are available from the corresponding author upon reasonable request.

## Supplementary Materials

The following are available online at https://www.mdpi.com/1422-0067/22/1/396/s1.

## Abbreviations

4CL	4-coumarate ligase
Cas9	CRISPR associated protein 9
CRISPR	Clustered regularly interspaced short palindromic repeats
GMO	Genetically modified organisms
gRNA	guide RNA
HDR	Homology-directed repair
NHEJ	Non-homologous end joining
NLS	Nuclear localization sequence
PAM	Protospacer adjacent motif
RNP	Ribonucleoprotein
sgRNA	Synthetic guide RNA
trcrRNA	tracer RNA
