# Supplementary material for: First Report of CRISPR/Cas9 Mediated DNA-Free Editing of 4CL and RVE7 Genes in Chickpea Protoplasts
Source: Int J Mol Sci. 2021 Jan 1;22(1):396. doi: 10.3390/ijms22010396 (PMC7795094; doi:10.3390/ijms22010396)
Supplement: Supplementary file 1 [file ijms-22-00396-s001.pdf]

First Report of CRISPR/Cas9 Mediated DNA-Free Editing of *4CL* and *RVE7* Genes in Chickpea Protoplasts

Sapna Badhan, Andrew S. Ball and Nitin Mantri *

The Pangenomics Lab, School of Science, RMIT University, Melbourne 3000, Australia; s3418286@student.rmit.edu.au (S.B.); andy.ball@rmit.edu.au (A.S.B.)
* Correspondence: nitin.mantri@rmit.edu.au; Tel.: +61-3-9925-7152 (ext. 7110)

Supplementary Data

Supplementary Table1: The PCR program to amplify the 5kb genomic region is provided in Supplementary Table 1.

PCR steps	Temperature	Duration
Initial Denaturation	95˚C	2 min
Denaturation	93˚C	30 sec
Annealing	52˚C	30sec
Extension	68˚C	5min
Final Extension	72˚C	10 min

Supplementary Table2: The PCR program for sequencing samples is also provided in Supplementary Table 2.

PCR steps	Temperature	Duration
Initial Denaturation	95˚C	1 min
Denaturation	95˚C	15sec
Annealing	55˚C	30sec
Extension	72˚C	10sec

Supplementary Table3: The PCR reaction to amplify the 5kb genomic region is provided in Supplementary Table 3

Component	Amount(μl)	Final Concentration
GoTaq® Long PCR Master Mix, 2X	25μl	1X
upstream primer	5–50pmol	0.1–1.0μM
downstream primer	5–50pmol	0.1–1.0μM
template DNA	0.1–0.5μg1	<0.5μg/50μl
Nuclease-Free Water to a final volume of	50μl	

Supplementary Table4: The PCR reaction to amplify the sequencing fragment is provided in Supplementary Table 4

**Component**	**Amount(µl)**
Template	200 ng
Primers (20 µM each)	1 µL
MyTaq Red Mix, 2x	25 µL
Water (ddH2O) up to 50 µL	50 µL

Supplementary material 1

Sequence used for 5kb target amplification for *4CL* gene

4-coumarate--CoA ligase-like 1

NW_004516753.1_162798-165892

Length 5094

ATATATATATATATATATACTCTTTTGTTTTGTATATAAAAAAATATTGAGTCAACAAAAATTAATGTATTTGATT
CTAAATTTAAATCATTTACTTTTAATATTTGAGAGTGGAAGGAGTATATGATTTTCTTATTCGTGAATTTTAAAA
TTTTAACACTTGAATTTAACGTTTTTTATTTTAGTCTATAAATTAAAAGAATTGTTGTTTTTAATTTTTTACGTTAA
CTTATTGGACCACATGATGTGATTTTATTAGTGATGTGAAACTCTGAAAGCAATATTTTTTTTTAATTTGCAGAG
A GTTTAGGTTACCGAACGAAGAGG
CGAGATTCAAATAAAAAAGTTATGAAAATTAAATAAAAAAACACTATAATTACGTGGAGTAAAATTATATTTA
AATAATAATATTATTTAATTTTTGACTAGTGGTTTGAAATTGAATGTATATAAAACGCGAGATGCATAGATGAC
TTTGTATTGGTTTGCAATTTGGTAGAGGGACGCAACAAATGTAATTTTGAAGTTGTTGAATGAACCTTCACCGA
AAAAATTGAAAAAGACCATTGATAACTGTAATCCAATTAAGGGAATTAATTTAATTTATATCGTCGTTGTAAAA
AAAAAAAGTGTAATGTCAATCAATTACAAATGATAAATTTTTAATAATAATTGACTTTTATCGTAATTATTTTTA
AAGTCATTCACATAATTGATTTTGATTTGTTGATTAAATAAAAAAAATTACATTGTTAATATATAAAAATTAAAA
ATTATACTCTTTAAAAAATATATATGCAACAATTCCATAGATTGTCCATTTTCTATGAGACTCATTCTTGACAAT
GCCACAAAAGATTATCATATATGTTTGTTCACTAACTTCAAATGGTAACTTATGTTAATTGCACCTAAAGTCATA
TTCTAAACTAGAATAATGGCTTTGTTTGATAGGTGCTAGACAACTTCATTTGAATCACAATAGTTTGACAGAAT
ATAACATAAAATATAATGACAAATCAAACATACCAACATATAATTATCAAATTTTATTCTCAAACACTAATTTCA
ACATATGCTAAAATAACATGATGTCAAATTAGCCATCACTTAACAACATTACAATACCAATGAACTAGACGGTG
ACATATGAAACCTCAATTAAAGCATATTATATAATTGTTTTTTACTAATTTGAAGATGTGTTGGATTTTGTTAGG
GAATTTTCCTTCATTTTTTCCACCATCTTTTCTTTGATAAGCCTTCTCATTATTTTACCAGAAGGTGATTTTGGTAT
AGCTTCCACAAAGTGAACCACTCTCACTTTCTTATAATGAGCAGCATTGGAAGCAACATAGTTCATAATGTCTT
GTTCACTCTCTTTTGCACCTATGCTTAAAACAACACTTGCTGCTGGGATTTCCCCTGCCTCTTCGTTCGGTAACC
TAAACAACGTTGCACAAAAACAACATAAAGGAAAATGTTAATTGTCACGAAACATTTTTATCACAAAAGCTAT
GTATTTTAATGTAATTGGTTTGAATTTTCAACTCAGACATAATGTGTATGATGTCGAACATAGAAAATGATGAT
TGATTGCTTACGGTACAACAGCTGCATCTTCGACCGACGAATGAGACAACAGAATAGCTTCTAATTCAGCTCA
GCAGGAGCAACCTACATTCATAAAATAAAATTAAATAAAAAGACACAAATTAATTTTAGTCTATATTGGATCAT
ATGAATATGCGATCCCTTTGACTTAATTTTCTTTTTAAAAAAAAATCATTTTTAAAATAAACTTACTTGGAAGCC
TTTATATTTAATCAACTCCTTAATACGATCAACAATAAAAACATTTTCTTCATCGTCTATGAATCCTACATCACCA
GTGTGAAGCCATCCATTGTTGTCAATAGTTTGAGCAGTCTCATCCACCTGTTTATAGTAACCTAGTAAAAACAC
AAATAGTTTATATAATTTAATCATAATTTTACAAATGGTTAATTTATGTGAAACATTATATATCTCAATTTCATTG
TACCTTGCATTACACATTGGCTCCTTACACAAAGTTCCCCTGGTGTGTTCCTTGGGAGAGATCTACCTGTATCA
GGATCAATAAACTTCACTTCCAAATTTGGAAGGATGAATCCAACTGAATTTTTATGTGTATTTCCATATCCTTTT

TGTGCATGTGTAAGTGTTATGCAACTATGTTCAGTTAGTCCATATGCCTGCAAAAGAAAAAATAGCACTTTAGA
AAAAAATCACATACTTTTTACCTAATACACAAACATGAAATTGTTTAGAAGGATGAAGTAATCACCTCTTGGAC
AAGAACACCAGGAAACTTGTGTTCAAAGGAAGTGAGTATTTCAGGTGCAAGTGGTGCTGCTGCAGTCATAAT
AGCTTGAAGTTTAAGCTTAGTGAGATCAAATTCATCCACAATAGGATTTTTAACTAATCCAAGAATAATAGGTG
GAACAATAGGTGCAAATGTGACCTCATGTGTTATCAAAGCATTCAAAAATGTTTTCAAGTCAAACCTTCCCATG
ACAACAACTTTCCCTTTGTTCCTAATAGTAGCACAACATATTCCAGTGATGCCATAAATGTGAAAAAATGGAAT
TAAGCCTAATGTTGTGACTTGACCAATCATTTCTTGTGCTACACCAAAAAGTGTGGAACAAAGATTTGCTACTA
GGTTTCTATGAGTGAGCATTACACCCTTTGACAATCCTGTTGTGCCTGATGAGAATGGCATGGCACAAAGATC
ATTTTGGTGAATTTGTTCCTTCATTGAGTCATCACCTGCCCTGTCTGCTGCTTCAAGAAGTTTGTTCCAATTCAT
AGCATCTTCAATTAGTTCATCTCCTAGTACAATAATTGGTAGCCCTAGATTTTTCACCTGTTTCAATTTCAACACA
AGAAGTGTCAAATTCTAGTTCAATGTGTTCAATTGTGGGTCTTGTTTGTTTCACAAAACATTTTTTGTTTTCACC
GTATGTTATAAATTTGTTACCTTGTTTTCGCTCTGATTCTCATTTTCAAATATTTATACAAGAAAACGTGAAAACT
GTATTTGTATTTTCATTTTTTCCTATATTAATTAAATGGTATGTCCTATTAGTGTAAAACCATTCAAAATTTATGA
ATACATCAATTCAATATTTGTATTTTTTTTATTTAAAAAAACGTATAATATGATGTGATAGATTGAACAATATAT
AGAATTTAATTTCAAATAAAAATAAAAACATATCAAATCAAACATACATTATTGGTCGGTTTGTATTGATTTATT
TGAATTGGTGAAAACAATTTATAATATGTCCATAAATTGTTTTCATAAATATTTTAAGATAATTTATGAAAACAA
CTTATATATAAACAATTTGTGTTTATTTTATTTTTATTATAGAAACAACGTATATACACAAAACACTTATAAAGT
ACATGATTTATCATTTTGGAAGAGTTACCTTTTCATGAGTTACACTATTTGTGACAATTAATTTGGCATCAGCAG
ACTCAGCTTGTTTCTTAATTTCTGAAGCATGCAAAGTTGGATTTGCACCTGAAAACACACCACCAGAATCCATA
ATCCCCAAAGCAACAATTGCATATTCAACAATATTTGGAAGCACTACAATCACAACATTCCCTTTTCTCAAACCA
AGAGATCTCAAAGCCTTTGAAAATCTATGTATATCTCTCTCAACTTCACTATATGTGACACATTTTCCACTCTCA
GCATCCACAAATGCAACTTTATCACCATACAATTCAACATTTTGTAGCACAAATTCTGGTAATGTCACATTGTCC
GGAACCGGAACAGGCGAGTATTGGCTACGAAAAATGTGTTCTTTCTCTTCTACAAAATTTTCAATGTAAGTTCC
CATGTTATGTTTTTTTCTTTTCAATGTAAGTTCCAATTGAAATGAATGAAGTTAAATAATGAGGTTGAAAATGT
GAGATTATATGGACAATTTTATTTAGTTTTTTAGTCATGGTCATACTTTTTGTATGGTTTGAGGTTAGAAATGTT
GTAAGTGAAGAGGATTTGAAAATTTGTCATGCATGTGGTTTTTGTTGGTGATAATTTGTTGATTCATGTTATTG
ACAATGGCCAAGTAAAATATTTGTGTAATATCTCTATTTTTGGTGAATGTGAATATTTTCCATTAACCAATAAAT
GTAGGTGATGCATTGCCTATGAAAGTTTTCTTGTTTCCATCCTAATATACTAAAAGGTAGAAGAGGTGTTGGT
GAAAGGTGACACTTTGAATTTTCTTTAATTTTCAGCTTTTAATATATGAATTGTAACACGATTGGATATTTTTTTC
AGAAGAATTCCAATTGGGATAAAACTTACTTTCAAAATTGAATTTGTGCGTGTGTATTTTATTCTTCATTTTAAT
TAAAAGTAATTTAAATATAAATAACTTATAGGGTTAAATAAGTACTTCCTTTTTATAAAATTTCAAAATTTTGTT
TTTAGTTCATGTACAAAACTTTTTTAATATTTTTTAGTTCATACAAAATTATTATGTAAATGGATTTTGTCTATAT
TAAAATATCGATATATATTTTTGAGTGATTTTTTACAGATATATTTACAATATTGTAAAATGTTCTTCCACAAAA
AACGAGTTTAAAGTTCGATTTTTAAGTTTATATTTTTGTTAGTGTTTTTATTTTGGTTTATGACATTTAAAAATTC
ATATTTAATTCATTTGAAGTTAAAAAAAGTCTAAATTTTTGTGGATAAATTTTTGAACTTATTTGCATAAAGATC
ATTCAAAAATAAGGCCAATTAAATATGTTTCTTTCCAGTAAGGACTAAACTATTTGTTGAAAAAATTTGTAGGA
AAAATAATTATAGTGCTTTTGCGGGACTAAGATAATTAAATATGAATTTAGAATTTAGAATCATAAACATTTAA
AATTAAATATAAAAATCATACCCATTTAGAATTTTTTTAATTTAAGATAATTAAATATGAATTTATTGAATGCCA
TCAACTCAACATAAAAATAACAAAAATATAGACCTAGAAATTGAATTTTAAATTACTTTTTGTGGAGGAACTTT
TTGCAATGTTATAATATGTCTATAAAAATTCATTCATAAATACATGTCAACATTCTAACAGAGAATAAAAATAG
TTGCATAATAATTTTGTAGGAATTAAAAAATGTTAATTTGTTTTACAATACTAAAATCAAAATTTTAAAATTTTA
TAGGAAAATGAGAAAAATCATTTGTACAAAAAAACTAAAACATAAAACAGTATAAGAAAATGAATTATTATTT
TTTTAATGTATGAATAAAAATATTTTTTCTTCATATATTAGTGATAAATATTAACTTTTTTTTTAAACAATGCTCA
ACAACATTACATAATATTCAACCAAGCAAAACAATGCTAGGAAACAAAAACGTTTCACACAATTAAGTTTCAAC
ACGATAATTTGAAATTGGGGAAATGAATTTCATTTTCTACATATTAGAGAATATTATGTTCAATACGAAAAGAT
GTTATTTTAGTGCTTTTTTAGTTTGAGCTATTCGTTTTTACGTTTATTTTTTATTTATGCGTAAAATTTAGGAGAG
CAATTCAAAATATATATTTTAATAATTAATAAAAGATGAAAATAAATGTGAATATGAATATAAAAAAATAGGA
ATAAAATTACACTAATGTAATGATATTTGATAATTTTTTAATTGATACCAATTTTACAAAATACACTATTTAATTG

AAAATTTATATCATATAAATCATTCATATCAAATTTGTACAAACCTACAGTCATTTGATTGATTATCGAGATTTA
CAGAGATTAACGACGTTTAAAAATAACATATAAGACATTAATTTTGATATATCTTAATAATATACCAAATATTTT
TATATT

Supplementary material 2

Sequence used for 5kb target amplification for *RVE7*

RVE7

NW_004516329.1_420654-425384

CTATTATAATATATACTTTTTTGTTAAAAATATAATCACTTTGTTTAAATAATAATAAAAAATAGCACTTGGACT
CAAACCCTAAATCTAATTAGAACAGCAAGTGAGATTCACATGCTATGACTGCGAGTGGAATAAAAAAATAAAA
AAAATGACAATAAGTCAAAAAAGAAACACTAAAAAACAAGAAAAAAATATCTTATCAGTTATCTTCTCTCAACT
AATGCCTTCCACAAATATTATCTGAAACACTACAAAATATTTAACCAATTATTTACTTTTTAATTTTTTTCAATA
AAAAAATCAGACATCACAGTTACGCGCCACGTGTCGTTTGTCTCAGAACATTTTCAGCCACAAAACTCAAACCC
TTTCTCGAACCCTCCATTGAAATTTCTTTTTATTTTCATTTTTATTTATTCGAAAATTTCTCAATCTGACGTGTCAA
GCTCTTATTTTTTCTTATTTATTTTAATTTTAATTATTTATTTATTTATTTATAATCTCTTTTTCTGTTCTTCTTTTTCT
CCCCTTCTCTGCTCAAACCTTCTTCTTTAGCCTCAATCTCTCTTTTCTCTCCAAATTCTTCATCTGGGTCTCTTTCAT
TTTCTCGGAAAATCTCTTTTTTCCGAGAAAGTTCACTTTTTTACTTCAAAATTGATACTAAAATAAATCGGTTGA
TTCCACTGAAATCACAAGGTATTTTTATTTTTATTTATTTGTTCCAAATTTTTATGTGTTTCATAAAAACAAATTT
CTTTTTGGATTTAGTTCAAAACATGTATTTAAGATTCAGAATTTGATTGATCTGATTTGTAAAAGGGTACTTTTG
TGTTGTTTTTTTGTTAGTTTCTATTTTTAGAATGTTCTATGAGCTTTTATTATATAAAAAAAGATTGTGAAAAATG
TTATGTGATTGTGATTTTCTTTTTATTTTGCTAAAAGATTTAAGATTCACTTTTAATTTTGGTTATTTTTTATTGGT
GGCAGTGAAGATTAAGGTGTTCTGGTCTTTGACTCTGATTTTGCTACAATGGAGATTAAGGTATATAACAAGG
AAAAGTTCAAACTTCAACCTTTTTTGAATACCCTTTTATGTTTTTTATCTTATAATTTCATTGTTTGAAAGAAGAA
AAAAAAAAGGTTGATTTTTATTTTGCTGAATTTTATGTAGTGATTTTTTGTTTGTAGAGTATTTATTTGAGTTTG
TTCTTTTTTGTGAAGAAACATGTGTTTTAGAAGCGTTTTAGCTGAAACAATTTTTTGTCAGACTTTACTTACCTTT
TAGAGAAATTCTAGATTAGTAGGGTCCCATTCCCTGGGAACTCGATGGTTTAAACCAAAATATGTGTTTTAGA
AGCTATGTGTTGTGTGTGGAGTATGCAAATTCCATAGGAGAGCTAAAAAAGTTTTATTAAGAATGAATTGGAA
GATTTCTGAATGGATTCATTAGTTAGATTCTGAAATAATAAATTGACATGGTAGTTGAGTTATAACTTGATCAG
CATCTAAGGTTAAGAAGCTGAAATTTTCTAATACTAATTACTAACATTAGCCGGTTTATAAAAAATAAAGTTGA
AATTTTCTGGTCAATCATTTTGTTTATGTGTTTCTTATTGTTTAGAGTACAGCTTAGGACTATCTCAATCAGGTC
AATTTGATTCATACTTCTAGTGATGTTGTGAGATTTTATTCTTAGGATAAAACTTAGGTCGAGTTGATTTAGTTA
AGTTCGCCGTGTTCTTCACTAATAATAGGCTAATATAATCACCAACTACTTTCACCTATTTTTTGAGGATGTATC
TTTTTATCGGATATTTTAAGTATTTTTTGCATTTTTTAGTGAAGAATATGACGAATTTGACCTAAGATTTATCCTT
ATTCTTATGTGGGGAAAGGTAGCTTAATTAGATCAATCTATATAAGCAAAAATGGCATCTTAAAAGTTGTTGTA
TTTGGTCTAAATTTTCGGACCAGATACAACAACTTTTTATATGCCAGTTTTGTTTATATTTCATTCCAGATGGAG
TATATCTTTTGATTCTGTGTAAATCCCCTTGAATGTTGGAGTGAGGTTACGAAGAAGATGTAACATTGAACCTT
GTAAACACCTCAGTTGATAATTTTCATGTATAAACATTATTTGACATAGTGTTCTTACTGAAATATCACTGCATT
TTTTGGTTTTTGTGAGTGTATGTATTTGCATGAGTTAGCTTGTGAGATTTGAAAGGGATGGCGATTAAGCTTAG
GTCTATTTAACATGCTGCTGCTTGGTTGGAAGCAGGTTTCATCCGTCTTACATTCAATCCTCCACTCTTCCGGAG
ACTTTTCTTCTCTTTTCATAGTAAAAGAGGGTTTCAATATGAATTCATCCAGTGACTTTATTTCTTATATCGAAAC
ATATTCTAGTGAGCATCAATTTCTTTCATCATGAAATTAGTCCCTCTCTTCGTCTTTTGTTTTTCGGTTTTTTTTT

GTTCTTGAACAGTTTTTAATTCATTAGTTACTTTTGAATGTAGAACCAAGTAGAAGGCACGAAATCGACTATAA
TCGAGACAGAAAGTAAGTGTCATTCCGAAGGTGGAGAACAGCCGGAAAATGTTGCGAAATCGCAGGATATAC
CTTCTGTTGGAAATGGAAACAACCTTACTCCCAAGGTAATTTTACTTTCCACAATCCATTTTGAAATATTGTTTTT
CCAATTGAAGAACTACCCTTTTGTTCCAATTTAACGTTTTGTTCGGAAACTTGTTTCGATTTTAAACAGGTGAGG
AAACCGTATACCATCACTAAACAAAGGGAGAAGTGGACTGACAAAGAGCATCAAAAGTTCGTCGATGCTTTG
AAATTGTATGGTCGTGGCTGGCGTCAAATTGAAGGTAAAATTTATGTACTATATTGGTTGAACTAATTTTACAA
TAAATGATTTTCCATTTCTTCAAGTTAGATAAAGTCCTCTTAATCTTCATGCAGAACACATAGGAACCAAAACC
GCGGTTCAGATTCGAAGCCATGCTCAAAAGTTTTTCTCTAAGGTTTATATTTTAATTTTCAGTACATAATTCGTG
TCTCTAAACTGAAGGTTACAAACATAGAGTTGATTTTAATTGGACTTTCAAGAAAGAGATTGATTTTGATACAG
TGTCAGTGTAAAACTTTTTACTCTGTCAATTAATCGTAGTTCGTCTGTTTGACTTTTAAAGTATATATTATTAAAA
TCAAATATTTTTAGTGATCGAACAACTGTGATTGATTGACAATGTTAAAAACCTTTACACCGTCGGTGTATATC
AATTAAATTCTTCTAGGATTATGATAATTGGCACTTTAATAACATAATAAAAGCGGAGATGTATTATTCTATTTC
AAGTTTCAACATTCCATGTATCGTAATATCTACTTGGTTACAGGTTGTGCGCGAACATGATGGCAGCGCCGAA
AGTTCTATTCAACCGATTGTCATACCTCCTCCTCGGCCTAAGAGAAAACCCCTTCATCCGTACCCCCGCAAATCA
GTTGACTCTATCAAAGGACAGCCCGTTCCAAACAAATCAGAAACATCTCCATCAGTTAATCTGTCAGTTGCAGA
AAATGACACTCAATCTCCAACCTCTGTACTATCTGCATTTGCTTCCGAAGCATTTGGGTCGGCAACATTTTCTGA
GCAGACTAACAGATGCCTTTCTCCAAATTCTTGCACCACCGAGACTCACCCGATCAACTTGTCGCCTGTTGAAA
AAGAAAATGATTGCATGACATCCAAACCATCTGAGGAGGGAGAGAAAGAATCTCTAGCCACAGTTCCTTTATC
TACCGACTCAAAGCCACTTATTTGCATGGTATGGTAAACTTCATGATATTCTACAATATATCTTCATAGTTCATA
GAACCCTTACTATATGTTTGTTTTCAAGGTGGAAAAATGCTAACCGTGCATTGAAACGAACACTCCAATATACT
ACTCCCTCCATAATGAAATATAAGTGAAAATGATAATTGAAAAGTTAATGTATCTGATCTTAATTTAAGATCAA
ATATATCAAATTTTAATTACTATTTTTGCTAATGTAGACGGAAAACAAACATGCATTTAGTCTATCACAGTTACA
GTTTTACTGATTTTGAAATTTTCTCGTGTTATCGCAGAAGTCCGAGATTAGTTCCTCTCAGGAAACTCAATGTTT
CAAAGAAGATGCTGCGAATAAACAACACATCACTAGTATCAAGCTGTTTGGTAGAACAGTCTCCATGATTGAT
AATCAGAAACCGATGAAGGAAGATGATGATGATAATACTAAGCCGATAACTATCAAATCTGATGATGAAACG
AACAATGTTGAGAATGAGAAAGTTGGTCAAGAAGGGATATCAGGGCAACTTGAAACACAATTATCGCTAACC
ATGTGTAATGGTATGGAAAACCCGAATGAAAATCAGTGCGTAGGCGAGTGTGCAGCTGATGTATCGAGGTGG
AGTTTGTACCAAGGCCTTCCAGCTGTCAACTTCAAGACATCCTGCAATCATCAGATTCTTAATCCTGTGCCACTT
AGGCCATTTTTGAAGGTAAGAACAAGAGAAGAAGAAAGTTCTTGCACTGGTTCTAATACTGAATCAGTTTGTG
ATAACACTGTTGATTCTCAAACTCAAAAACATCATCAGAAATCAGGGAGAGGGTTTGTACCATATAAAAGATG
TTTAGCTGAAAGAGATGAAAATTCTTTAATTGTTGCTTTTGAAGAGAGAGAGGGACAAAGAGCTCGCGTTTGT
TCATAATGGACCCGGATTCCCTGCAGTAAGAAGTTCACTCACGAAATTTTGCGCAGTTGTTAATCTCAATCATT
AATCTGAGATCAGACGGTTTAGATCGAAAACTGTGTCAATTAAAATGATCTCGACGGTTGATGTGAATTCAGA
CGACTTAAGTCGGTTACATCATCATGAATAACTGCAGGAAATCCGATTCTGTTCATAATAGCTTTTCTGTTCCAA
TTTTTTGTTTACATGGTTTCATTGTGTTTGTTAAAAAAAACTTCCATAGAAATAAGGAACTGGTTTAGGTAGCT
GTGATCTAATTGTCAAGTATTTGATTGTCAATTCTTTTTGCAATGTTTTCTACATGATCTTTGATCTTCTGTAATA
CCTTCTTCCACACTTTGATGTCCTATAATTCTTCTGCTGTATCATTTTACAGTTTAACCAAATAGGGACACATACC
AGCAAATCTTTTGTTAATTTCTATATCTTTCTTTTCCCTCCATTTTTGGAATCTATTCTTTATAGTCGCATGATTGG
ATCAATGATTGAGGCACATAAAGGAAGCATGGATAAGCAAACCATGTCACATAACATAATTGGATCATTAATC
TCAAAAACTTGAATGCCTGGATTTTCTTCTATATCCAATCATTTTATCGGTAAATTTTAACGACATTCATCAATAC
TTTTCAGAAGAATTAGTTCATGATCCTTTTTGTCATTATAAATAAAACGATTTAATTAATGTTGATTTCATACAA
AATATATATTTTTATTTTATTTTTATTAACACTAATATTTTAAGTCATTATTTCAGTCATGAAAATTATGAAGATG
ACCATATATGTTTATAATTGTTGAAATTTTAAATTTTTTTAAATACTAGTCCATGATGGATCGATCTGTTTCTTCC
TTGTTGAATTTGCTGGATGATTGGAACCAAAAGATTGTAGTCCACTTCTATTATACATTATGTTTCCTAATAATA
ATTTAATAAGAACAATGTTACTATCATATGGAAAGAACCATCATATATAGTATCAGAGTACAATGCAGAAAGT
ATTAAGACAAATTCTACTGT
